# Quantifying Particle and Interaction Effects of Crumb Rubber on Asphalt Rheology at High Temperatures

**DOI:** 10.3390/ma19061085

**Published:** 2026-03-11

**Authors:** Shanwei Li, Xiaokang Zhao, Jiupeng Zhang, Fusen Zheng, Shuxian Zhang

**Affiliations:** 1 School of Highway, Chang’an University, Xi’an 710000, China; 2 Shanghai Municipal Engineering Design Institute (Group) Co., Ltd., Shanghai 200000, China

**Keywords:** particle effect (PE), interaction effect (IE), CRMA, filtration procedure, high-temperature rheological performance, GRA

## Abstract

To evaluate the respective contributions of the crumb rubber (CR) particle effect (PE) and the CR–asphalt interaction effect (IE) to the high-temperature rheological performance of crumb rubber modified asphalt (CRMA), a CR filtration approach was designed to physically separate CR particles from CRMA. Fluorescence microscopy (FM), dynamic shear rheometer (DSR) tests, and gray relational analysis (GRA) were conducted on CRMA binders with different CR particle sizes and contents before and after filtration. The results indicate that the retained CR ratio ($R_{\mathrm{cr}}$) increased with the increasing CR particle size and content, and coarser CR and higher CR contents generally increased G* and decreased *δ*, indicating enhanced high-temperature deformation resistance and recoverable deformation capacity of CRMA. After filtration, G* decreased markedly, whereas *δ* increased, and the quantified PE and IE results further indicate that the enhanced high-temperature rheological performance is dominated by PE, with IE providing an additional contribution, particularly at higher CR contents. Moreover, $R_{\mathrm{cr}}$ correlated positively with G* and negatively with *δ*, and GRA suggested that CR content acts as the primary factor affecting high-temperature performance, while CR particle size serves as a secondary factor. Overall, this study provides practical guidance for optimizing CRMA design and supports improved asphalt service performance.

## 1. Introduction

With the growing emphasis on sustainable transportation and green infrastructure, the recycling and reuse of end-of-life tires has become an important research topic in road engineering materials [1,2]. End-of-life tires can be processed into crumb rubber (CR), which is used to prepare crumb rubber modified asphalt (CRMA), where enhanced CR–asphalt interactions contribute to improved pavement performance [3,4]. In practice, CRMA can be prepared via two routes: the dry process [5] and the wet process [6]. Beyond these two routes, terminal blend CRMA have also been widely studied to improve storage stability while maintaining performance benefits [7]. Current specifications in China predominantly adopt the wet process for CRMA preparation, where CR–asphalt interactions are mainly influenced by the physical swelling of CR and the partial devulcanization and depolymerization of CR within the asphalt phase [8]. CRMA prepared via this method generally exhibits enhanced high-temperature rutting resistance and improved elastic recovery behavior [9,10]. Studies in U.S. and Europe have also reported that wet process CRMA can improve rutting resistance and deformation recovery, while the magnitude of benefit depends strongly on CR contents, particle size, and blending route [11].

The CR–asphalt interactions can be described in two contributions: the particle effect (PE) and the interaction effect (IE) [12,13]. With advances in characterization tools and testing techniques, researchers have reported that complex chemical reactions may occur between CR and asphalt during CRMA preparation; however, the associated performance enhancement is still mainly attributed to physical interactions between CR and asphalt [14,15]. CRMA is a heterogeneous composite system in which rubber inclusions and their swollen morphology can generate a particle-driven structure that influences asphalt stiffness and viscoelastic response, particularly at high rubber contents [16]. PE refers to the formation of the interlocked skeleton within the asphalt matrix, which increases the asphalt stiffness and high-temperature deformation resistance [17]. Such skeleton-like reinforcement is commonly described as a particle-driven load-bearing framework and becomes more pronounced when particle contacts and agglomeration increase, thereby strengthening its resistance to permanent deformation at high temperatures [13,18]. IE refers to swelling and interfacial interactions between CR and asphalt, which enhance CR–asphalt compatibility and improve rheological behavior [19].

Wang et al. reported that the swelling of CR particles in CRMA depends on the CR properties (e.g., CR particle size and content) [20,21]. During CR modification, the CR–asphalt interaction effect can be described as a diffusion and swelling process, in which CR particles absorb aromatic fractions from the asphalt, resulting in CR particle swelling and volume expansion. This swelling-driven interaction is also widely accepted to increase binder viscosity and elastic response, because the swollen rubber phase reduces the effective free light fractions in asphalt and alters the phase continuity [22]. Meanwhile, studies have indicated that CR particle size influences the extent of CR swelling and the effective CR–asphalt interfacial area, whereas CR contents dictate particle contact frequency and internal network formation, jointly affecting rutting resistance and elastic recovery [23,24]. Ghavibazoo et al. suggested that components of CR (e.g., carbon black) may gradually diffuse into the base asphalt; upon coupling between CR and asphalt phases, a three-dimensional gel network may form, thereby enhancing the asphalt’s performance [25]. Based on filtration experiments, Putman et al. further indicated that performance improvements in CRMA are jointly influenced by the CR particle effect and CR–asphalt interactions [26,27]. Meanwhile, many studies have emphasized that CR particle sizes and contents are among the most critical internal factors influencing high-temperature rutting resistance, fatigue response, and storage stability of CRMA [28,29,30]. Li et al. reported that the absorption of asphalt light fractions by CR particles plays an important role in CRMA performance [31]. Moreover, Singh et al. further examined the effects of CR particles on the rheological behavior of asphalt [32]. However, the mechanisms and the respective contributions of the CR particle effect and CR–asphalt interaction effect remain unclear.

To address this issue, a CR particle filtration test was designed in this study to separate CR particles from CRMA. Fluorescence microscopy (FM) imaging was then employed to examine the microstructural distribution of CR particles within the asphalt matrix, and a dynamic shear rheometer (DSR) was conducted to evaluate the high-temperature rheological response of CRMA with different CR particle sizes and contents. Gray relational analysis (GRA) was finally performed to identify the key factors related to the high-temperature rheological performance of CRMA, providing a quantitative basis and clearer understanding for optimizing CRMA design and enhancing its service performance.

## 2. Materials

### 2.1. Asphalt

In this study, Donghai 70# asphalt (DH70) was selected as the base asphalt, which was supplied by Maoming Weilong Petrochemical Co., Ltd. (Maoming, China). The technical properties of DH70 were determined in accordance with the JTG E20-2011 [33], and the results are listed in Table 1.

### 2.2. Crumb Rubber

Crumb rubber (CR) was selected as the asphalt modifier in this study, which was obtained from the shredding of scrap tires. Three CR particle sizes were used: 40 mesh (0.425 mm), 60 mesh (0.250 mm), and 80 mesh (0.180 mm). The technical properties of CR were determined in accordance with the JT/T 797-2019 [34], and the results are listed in Table 2.

### 2.3. Preparation of CRMA

DH70 base asphalt was placed in a thermostatic oven at 150 °C for 60 min, and then transferred to an oil bath and heated to 180 °C. Crumb rubber modified asphalt (CRMA) was prepared by two-stage mixing, using a high-speed shear machine. First, CR was gradually added under low-speed mixing at 1000 r/min, and the CR content was 10%, 15%, and 20% of the weight of the asphalt, respectively. After CR addition, asphalt and CR mixtures were mixed at 3000 r/min for 35 min, and then CRMA for testing was obtained. In this study, nine CRMA binders considering different CR particle sizes and contents were prepared, following the above procedure. CRMA was labeled using the format of CR particle size and content: for example, 40-15 denotes CRMA with 40 mesh CR at a CR content of 15%.

### 2.4. Preparation of Filtered CRMA

**Figure 1:**
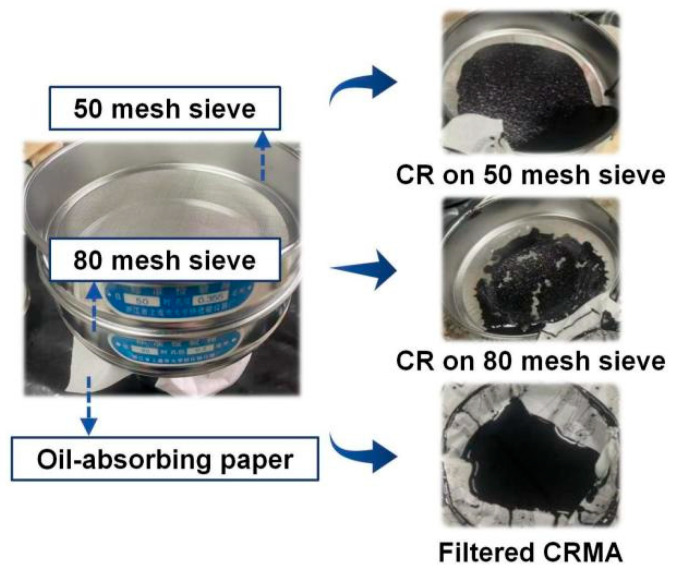
CR particle filtration apparatus and filtration products.

To investigate the respective contributions of the CR particle effect (PE) and the CR–asphalt interaction effect (IE) to the high-temperature rheological performance of CRMA binders, a CR particle filtration procedure was conducted using a two-layer standard sieve (50 mesh sieve on the upper layer and 80 mesh sieve on the lower layer), which can help to improve filtration stability and repeatability. The two-layer sieve was fixed on an empty steel cup, with an oil-absorbing paper interlayer placed between the sieve and the steel cup (Figure 1). This physical separation method can avoid potential chemical reactions that may occur in reagent-assisted separation methods, thereby avoiding confounding subsequent tests. After CRMA preparation, the hot CRMA was poured into the two-layer standard sieve assembly, and then placed in a thermostatic oven at 150 °C for 60 min. After filtration, the filtered CRMA was collected in the steel cup, while the unswollen CR particles and a small amount of residual asphalt were retained on the sieve surfaces. All nine CRMA binders were processed by following the filtration procedure described above, and the filtered CRMA binders were denoted by appending “-F” to the original CRMA label. For example, 40-10-F represents filtered 40-10 CRMA.

## 3. Methods

### 3.1. Filtration Weighing Method

After the filtration procedure, CR particles retained on the sieve were collected and wrapped with a 200 mesh screen. The wrapped CR particles were then immersed in dichloromethane (DCM) for 30 min to remove the gel layer adhering to the CR particle surfaces. Subsequently, the CR particles were taken out, air-dried to a constant mass, and weighed. Equation (1) was used to calculate the retained CR ratio ($R_{\mathrm{cr}}$), which is also used as an indicator of filtration (retention) efficiency.

(1) $$R_{\mathrm{cr}}=\frac{m_{f}}{m_{0}}\times 100\%$$

where $m_{0}$ is the total mass of CR used to prepare the CRMA binder and $m_{f}$ is the mass of CR retained on the sieve after filtration.

### 3.2. Fluorescence Microscopy

Fluorescence microscopy (FM) enables clear discrimination between DH70 base asphalt and the CR particle phase, based on the fluorescence contrast under the selected excitation wavelength, thereby allowing for characterization of the microscale distribution of CR particles in CRMA [36,37]. In this study, FM imaging was conducted using an LW300LFT-LED fluorescence microscope (Shanghai, China) on 40-10, 40-10-F, and 40-20 CRMA binders to provide microstructural evidence for enhanced high-temperature rheological behavior. The binders were heated to a flowable state, uniformly spread onto glass slides, cooled to room temperature, and then imaged. Because CR particles contain carbon black and other light-absorbing components, they exhibit negligible fluorescence and thus appear as dark spots in the FM images. Overall, FM is well suited for microstructural characterization of CR particle distribution within CRMA.

### 3.3. High-Temperature Rheological Tests

To evaluate high-temperature rheological performance, temperature-sweep (TS) tests were performed on DH70 and its CRMA binders with different CR particle sizes and contents before and after filtration to determine the complex shear modulus (G*) and phase angle (*δ*) [38]. TS tests were conducted using a SmartPave 102 dynamic shear rheometer (DSR; Anton Paar, Graz, Austria) with a 25 mm parallel plate and a 1 mm gap. The tests were carried out at an angular frequency of 10 rad/s with a strain amplitude of 10% over a temperature range of 40–80 °C, and G* and *δ* were recorded at 58, 64, 70, and 76 °C. Each sample was tested in duplicate, and the reported G* and *δ* values represent the average of two measurements.

### 3.4. Quantification of PE/IE Contributions

The high-temperature performance of CRMA is influenced by both the particle effect (PE) and the interaction effect (IE). PE reflects the contribution of the CR particle phase to the enhanced high-temperature performance, whereas IE represents the contribution from CR–asphalt interactions in the filtered CRMA binder. PE and IE were quantified using Equations (2) and (3).

(2) $$\begin{array}{c} PE=\left|\frac{P_{U}-P_{F}}{P_{0}}\right|\times 100\% \end{array}$$

(3) $$\begin{array}{c} IE=\left|\frac{P_{F}-P_{0}}{P_{0}}\right|\times 100\% \end{array}$$

where $P_{0}$, $P_{U}$, and $P_{F}$ represent the high-temperature rheological indices measured for DH70 base asphalt, unfiltered CRMA, and filtered CRMA, respectively. The index refers to either G* or *δ.*

### 3.5. Gray Relational Analysis

Gray relational analysis (GRA) is a system-theory method that is suitable for evaluating performance and evolutionary trends with limited and uncertain data. By quantifying the gray relational parameters among sequences, GRA helps to identify dominant factors and reveal underlying mechanisms driving these performance variations. In this study, GRA was employed to investigate the effects of CR particle sizes and contents on the high-temperature performance of asphalt. Because the high-temperature performance depends on CR’s particle size and content, their respective contributions were ranked using the average gray relational coefficient to identify the dominant factor [39]. The main steps of GRA are as follows:

Step 1: Define G* and *δ* as the reference sequences $x_{1}$ and $x_{2}$, respectively, and $R_{\mathrm{cr}}$, CR particle size and content as the comparative sequences $x_{3}$, $x_{4}$ and $x_{5}$, respectively.

Step 2: Normalize the G*, $R_{\mathrm{cr}}$, CR particle size and content sequences using Equation (4), whereas normalize the *δ* sequence using Equation (5), thereby scaling all sequences to the interval [0, 1] and yielding dimensionless sequences.

(4) $$x_{i}^{*}\left(k\right)=\frac{x_{i}\left(k\right)-\underset{k}{\min}x_{i}\left(k\right)}{\underset{k}{\max}x_{i}\left(k\right)-\underset{k}{\min}x_{i}\left(k\right)}$$

(5) $$x_{i}^{*}\left(k\right)=\frac{\underset{k}{\max}x_{i}\left(k\right)-x_{i}\left(k\right)}{\underset{k}{\max}x_{i}\left(k\right)-\underset{k}{\min}x_{i}\left(k\right)}$$

where $x_{i}\left(k\right)$ denotes the original value of the k-th indicator for the i-th sequence, and $x_{i}^{*}\left(k\right)$ denotes the corresponding normalized (dimensionless) value; $\underset{k}{\max}x_{i}\left(k\right)$ and $\underset{k}{\min}x_{i}\left(k\right)$ represent the maximum and minimum values within the i-th sequence, respectively.

Step 3: Calculate the absolute difference sequence $\Delta_{i}\left(k\right)$ between the reference sequence and each comparative sequence, using Equation (6).

(6) $$\Delta_{i}\left(k\right)=\left|x_{j}^{*}\left(k\right)-x_{m}^{*}\left(k\right)\right|,\;j\in \left\{1,\;2\right\},\;m\in \left\{3,\;4\right\}$$

where $x_{j}^{*}\left(k\right)$ and $x_{m}^{*}\left(k\right)$ denote the normalized values of the k-th indicator for the reference and comparative sequences, respectively.

Step 4: Calculate the gray relational coefficient between each reference sequence and each comparative sequence using Equation (7).

(7) $$\zeta_{i}\left(k\right)=\frac{\underset{i,\;k}{\min}\Delta_{i}\left(k\right)+\rho \underset{i,\;k}{\max}\Delta_{i}\left(k\right)}{\Delta_{i}\left(k\right)+\rho \underset{i,\;k}{\max}\Delta_{i}\left(k\right)}$$

where ρ is the distinguishing coefficient (ρ = 0.5).

Step 5: Calculate the gray relational grade for each comparative sequence, using Equation (8).

(8) $$r_{i}=\frac{1}{K}\sum_{k=1}^{K}\zeta_{i}\left(k\right)$$

where K is the number of data points in each sequence.

## 4. Results and Discussion

### 4.1. Effects of CR Particle Size and Content on $R_{\mathrm{cr}}$

**Figure 2:**
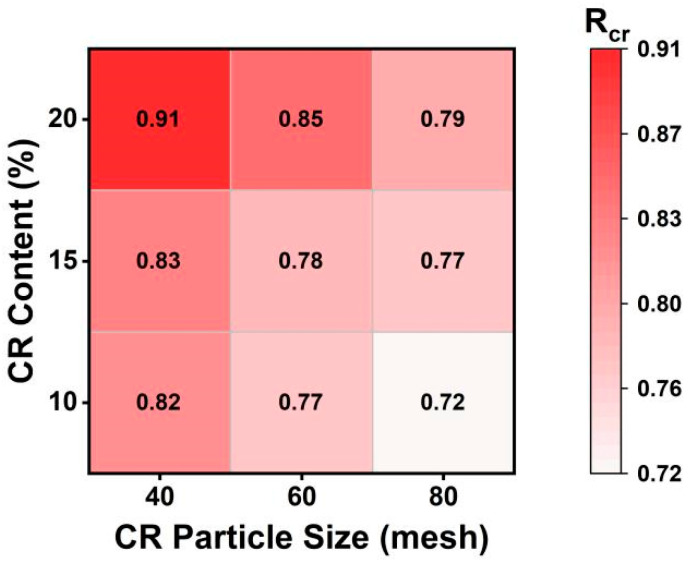
Heatmap of $R_{\mathrm{cr}}$ for CRMA binders with different CR particle sizes and contents.

Figure 2 presents a heat map of $R_{\mathrm{cr}}$ for different CR particle size–content combinations obtained from the filtration weighing method. Overall, $R_{\mathrm{cr}}$ increased with the increasing CR content at a given CR particle size; similarly, at a given CR content, coarser CR particles generally exhibited higher $R_{\mathrm{cr}}$, indicating higher filtration efficiency under the specified filtration conditions. This trend can be attributed to the lower specific surface area of coarser CR, which slows the swelling rate of CR particles and increases the CR mass retained on the sieve, indicating that coarser CR particles have greater potential to enhance particle-related contributions to high-temperature performance. To further clarify the relative contributions of the CR particle effect and the CR–asphalt interaction effect considering different CR particle sizes and contents, FM imaging and TS tests were conducted for verification and discussion in the subsequent sections.

### 4.2. CR–Asphalt Morphology Characterization

**Figure 3:**
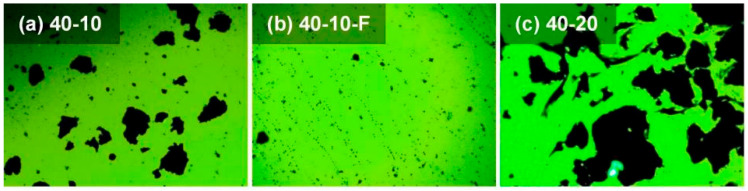
FM images (100×) of CRMA binders: (**a**) 40-10; (**b**) 40-10-F; and (**c**) 40-20.

FM imaging was conducted on 40-10, 40-10-F, and 40-20 CRMA binders to characterize the CR–asphalt microstructural distribution and morphology (Figure 3). Overall, CR was uniformly dispersed within the asphalt matrix, with no evident large-scale agglomeration or phase separation, indicating that the two-stage mixing method yields CRMA with a homogeneous microstructure and favorable dispersion. Image-based area estimation further shows that the dark-phase area fractions in Figure 3a–c are 12.9%, 1.2%, and 36.4%, respectively, suggesting a substantial reduction in the CR particle coverage area after filtration, whereas a higher CR content results in a markedly increased CR particle coverage area.

Comparing Figure 3a,b, CR particle coverage areas decreased by approximately 90.8% after filtration, indicating the effectiveness of the filtration process in removing CR particles. Accordingly, only a small amount of fine, uniformly distributed swollen CR particles was observed, while the asphalt phase remained continuous, indicating that most CR particles were removed during filtration. This agrees with the $R_{\mathrm{cr}}$ measured for 40-10 CRMA (Figure 2), suggesting limited residual particles in the filtered CRMA and thus a weak particle-related contribution [40]. Comparing Figure 3a,c, the increasing CR content increased the particle contacts and local agglomeration, promoting a more developed interlocking particle skeleton. Overall, the FM images provide direct microstructural evidence for CR dispersion and local agglomeration with different CR particle sizes and contents.

### 4.3. High-Temperature Rheological Performance

#### 4.3.1. Effects of CR Particle Size on G*

**Figure 4:**
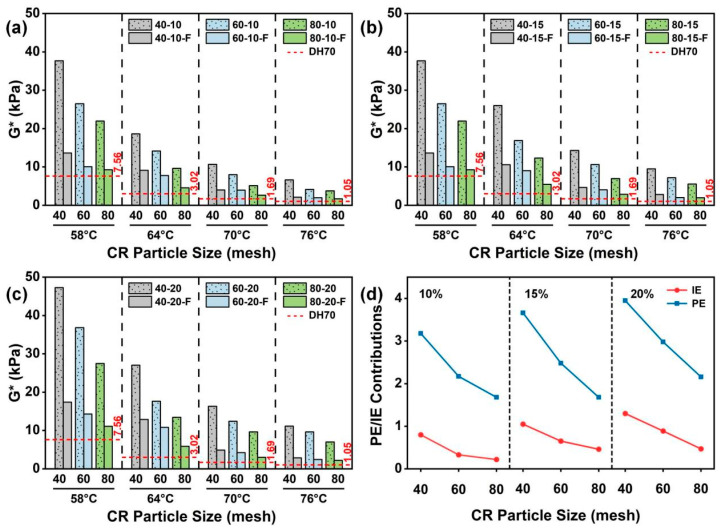
G* of CRMA binders with different CR particle sizes at a given CR content: (**a**) 10%; (**b**) 15%; (**c**) 20%; and (**d**) quantified PE and IE contributions at 58 °C.

Figure 4a–c show the effects of CR particle size on the G* of CRMA at a given CR content. Overall, all CRMA binders exhibited markedly higher G* than DH70 base asphalt at 58, 64, 70 and 76 °C, indicating that CR modification substantially enhanced the high-temperature stiffness and deformation resistance of asphalt. In addition, G* decreased with the increasing temperature, with a sharper drop from 58 to 64 °C and a more gradual decline from 64 to 76 °C. This trend is attributed to reduced asphalt viscosity and the softening and stress relaxation of the CR phase, which weaken the CR particle-related interlocking skeleton and thereby lower G* [41]. Specifically, at a given CR content, coarser CR particles led to a more pronounced increase in G*, suggesting that coarser CR is more favorable for forming a continuous interlocked particle skeleton and thereby enhancing high-temperature stiffness and deformation resistance.

After filtration, the G* of CRMA decreased markedly but remained higher than that of the DH70 base asphalt, suggesting that the improvement in high-temperature performance arises from the combined contributions of the CR particle effect and CR–asphalt interactions, with the particle effect as the primary contributor. This is primarily because filtration reduces the amount of CR particles, making it difficult to sustain an effective interlocked particle skeleton and thereby causing a significant drop in G*. Nevertheless, residual fine particles and swollen CR can still provide partial viscoelastic enhancement. Therefore, the filtered CRMA remains a higher G* than the base asphalt.

Figure 4d presents the quantified PE and IE at 58 °C. The results indicate that the increase in G* was dominated by PE, while IE provided an additional synergistic contribution. At a given CR content, both PE and IE decreased with the decreasing CR particle size, suggesting that coarser CR is more favorable for developing an interlocked particle skeleton and thus improving asphalt high-temperature stiffness. In contrast, although finer CR has a higher specific surface area and is more conducive to swelling and wetting, it mainly provides localized viscoelastic strengthening and contributes less to building a continuous interlocked particle skeleton. Therefore, its contribution to G* is relatively limited. Overall, G* decreased with the increasing temperature, and at a given CR content, coarser CR particles were more effective in maintaining the interlocked skeleton at high temperatures, thereby improving the high-temperature deformation resistance of asphalt.

#### 4.3.2. Effects of CR Content on G*

**Figure 5:**
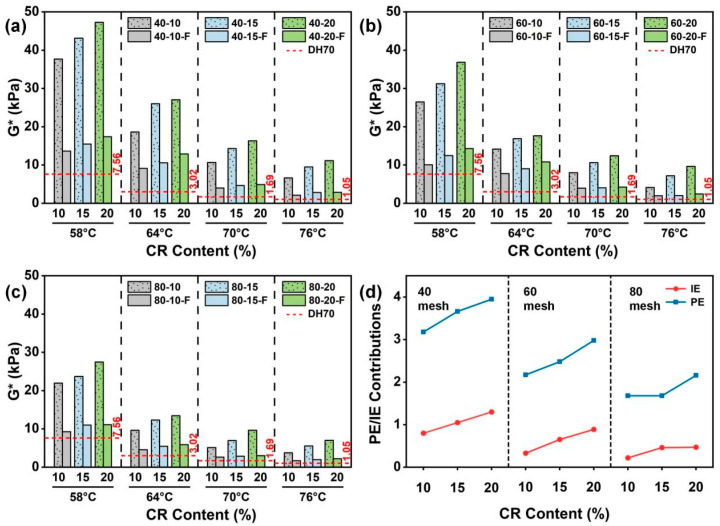
G* of CRMA binders with different CR contents at a given CR particle size: (**a**) 40 mesh; (**b**) 60 mesh; (**c**) 80 mesh; and (**d**) quantified PE and IE contributions at 58 °C.

Figure 5a–c show the effects of CR content on the G* of CRMA at a given CR particle size. Overall, G* increased with the increasing CR content, which can be attributed to a higher particle volume fraction and the higher probability of effective particle contacts, which facilitates the formation of a continuous interlocked particle skeleton, thereby enhancing high-temperature stiffness and deformation resistance.

Figure 5d further exhibits the quantified PE and IE with different CR contents at a given particle size at 58 °C. The results show that both PE and IE increased with the increasing CR content. On the one hand, higher CR contents increase the probability of particle contacts, making it easier to develop and sustain an effective interlocked skeleton, which effectively strengthens PE and improves asphalt high-temperature stiffness. On the other hand, a higher CR content allows for more particles to swell and interact with asphalt, enhancing the viscoelastic response of the asphalt and thereby strengthening CR–asphalt interactions and increasing IE. Overall, at a given particle size, moderately increasing the CR content is an effective approach to enhance the high-temperature deformation resistance of CRMA.

#### 4.3.3. Effects of CR Particle Size on δ

**Figure 6:**
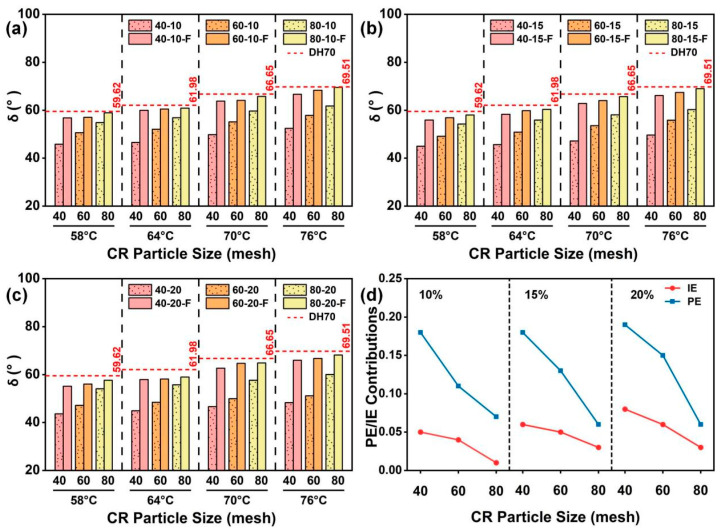
*δ* of CRMA binders with different CR particle sizes at a given CR content: (**a**) 10%; (**b**) 15%; (**c**) 20%; and (**d**) quantified PE and IE contributions at 58 °C.

Figure 6a–c show the effects of CR particle size on *δ* of CRMA at a given CR content. The results indicate that at all tested temperatures, CRMA exhibited lower *δ* than the DH70 base asphalt, and the reduction became more pronounced as the CR particle size increased, suggesting that CR modification enhances the elastic response of the asphalt, which is beneficial for improving the high-temperature recoverable deformation capacity [15]. With the increasing temperature, *δ* generally increased; however, at each temperature, *δ* consistently decreased with increasing CR particle size, indicating that CRMA with coarser CR particles maintained a lower *δ* at high temperatures and exhibited a more pronounced elastic response.

After filtration, *δ* increased for all CRMA binders but remained lower than that of the DH70 base asphalt, indicating a weakened particle effect while the interaction effect was partially retained. The increase (around 25%) at 58 °C was more pronounced for CRMA with coarser CR, indicating that filtration disrupted the interlocked particle structure and the associated viscoelastic reinforcement contributed by the CR particles. Nevertheless, the filtered CRMA still exhibited a lower *δ* than the base asphalt, suggesting that the residual fine, partially swollen CR particles in the asphalt matrix could still provide partial viscoelastic reinforcement; thus, some elastic advantage remained after filtration.

Figure 6d further presents the quantified PE and IE on *δ* at 58 °C, considering different CR particle sizes at a given CR content. The results show that the reduction in *δ* was still dominated by PE, while IE provided an additional synergistic enhancement. Moreover, at a given CR content, both PE and IE decreased as the CR particle size decreased, suggesting that coarser CR is more favorable for forming a stable interlocked particle structure and providing a stronger structural constraint. Consequently, coarser CR more effectively lowers *δ* and enhances the elastic response of CRMA. Overall, at a given CR content, using coarser CR particles is more beneficial for lowering *δ* and optimizing the high-temperature viscoelastic balance, thereby improving the high-temperature performance of asphalt.

#### 4.3.4. Effects of CR Content on δ

**Figure 7:**
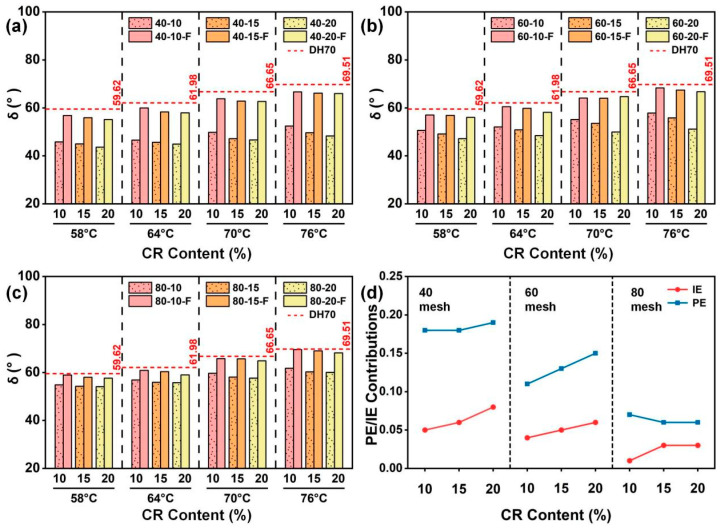
*δ* of CRMA binders with different CR contents at a given CR particle size: (**a**) 40 mesh; (**b**) 60 mesh; (**c**) 80 mesh; and (**d**) quantified PE and IE contributions at 58 °C.

Figure 7a–c compare the effects of the CR content on *δ* at a given CR particle size. The results show that *δ* decreased as the CR contents increased, indicating that the increasing CR content improved the high-temperature recoverable deformation capacity. This is mainly because the increasing CR content raised the probability of effective particle contacts, which facilitated the formation and maintenance of a continuous particle interlocked structure. Meanwhile, more CR swelling can further promote CR–asphalt interactions.

Furthermore, Figure 7d shows the respective contributions of PE and IE to the change in *δ* at 58 °C for CRMA binders with different contents. The results exhibited that the *δ* response was dominated by PE while IE provided an additional synergistic gain. Specifically, at a given CR particle size, PE exhibited no pronounced variation with the increasing CR content, indicating that PE is relatively insensitive to changes in CR content. In contrast, IE increased with the increasing CR content, suggesting that CR content-driven changes in *δ* are mainly attributable to enhanced CR–asphalt interactions. Overall, at a given CR particle size, moderately increasing the CR content can effectively decrease *δ*, thereby improving the high-temperature recoverable deformation capacity and rutting resistance of CRMA. Notably, comparing Figure 5d and Figure 7d shows that PE is more sensitive to CR content when evaluated by G*, because higher CR contents increase the volume fraction of swollen particles that provide mechanical reinforcement and restrict deformation, thereby strengthening G* more markedly [38,42]. In contrast, *δ* represents the relative balance between elastic and viscous responses [43]; with an increasing CR content, these contributions often grow concurrently, so their ratio changes only slightly, making *δ*-based PE weakly dependent on CR content [18].

### 4.4. GRA Results

#### 4.4.1. Correlation of $R_{\mathrm{cr}}$ with G* and δ

**Figure 8:**
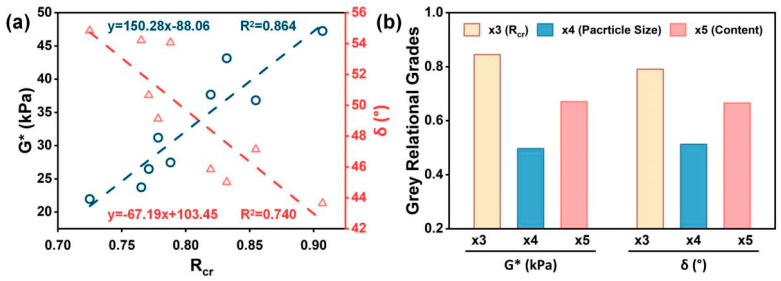
Correlation and GRA results: (**a**) linear regression fits of $R_{\mathrm{cr}}$ with G* and *δ* and (**b**) gray relational grades.

To elucidate the relationship between the retained CR ratio ($R_{\mathrm{cr}}$), based on the filtration weighing method, and the high-temperature rheological parameters of asphalt (G* and *δ*), linear regression analyses were performed between $R_{\mathrm{cr}}$-G* and $R_{\mathrm{cr}}$-*δ* at 58 °C (Figure 8a). The results indicate that $R_{\mathrm{cr}}$ exhibited a strong positive correlation with G* ($R^{2}=0.864$), indicating that a higher $R_{\mathrm{cr}}$ corresponded to a larger G*, suggesting increased asphalt stiffness and enhanced resistance to high-temperature deformation. Meanwhile, Figure 8a shows a negative correlation between $R_{\mathrm{cr}}$ and *δ* ($R^{2}=0.740$), suggesting that a higher $R_{\mathrm{cr}}$ corresponded to a lower *δ* and the stronger elastic response, which is favorable for enhancing the recoverable deformation capacity of asphalt at high temperature. Considering that G* is a larger-is-better indicator, whereas *δ* is a smaller-is-better indicator, $R_{\mathrm{cr}}$ was treated as a larger-is-better indicator for high-temperature rheological performance in this study. Accordingly, $R_{\mathrm{cr}}$ was then normalized using Equation (4) to support the following gray relational analysis.

#### 4.4.2. Dominant Factor Identification

To clarify the dominant factors associated with the high-temperature rheological response of CRMA, considering different CR particle sizes and contents, GRA was conducted to quantify the association between each comparative sequence and the reference sequence. As shown in Figure 8b, with G* as the reference sequence, the gray relational grades followed this ranking: $R_{\mathrm{cr}}$ (0.845) > CR content (0.671) > CR particle size (0.497). A similar ranking with *δ* as the reference sequence was obtained: $R_{\mathrm{cr}}$ (0.791) > CR content (0.666) > CR particle size (0.513). Notably, $R_{\mathrm{cr}}$ exhibited the highest gray relational grade for both reference sequences, indicating the strongest association with the high-temperature rheological response. As a filtration-driven indicator influenced by the CR particle size and content, $R_{\mathrm{cr}}$ more directly reflects the particle-retention state of CR within the asphalt matrix, and can serve as a laboratory indicator to assess and interpret the high-temperature rheological performance of CRMA. Overall, the CR content showed a stronger association with a high-temperature rheological response than the CR particle size, suggesting that CR content acts as the primary factor, while CR particle size serves as a secondary contributing factor to the high-temperature performance of CRMA.

## 5. Conclusions

To evaluate the contributions of the CR particle effect and CR–asphalt interactions to the high-temperature performance of CRMA, a CR filtration test was conducted to separate CR particles from CRMA. FM imaging was used to characterize the distribution and morphology of CR particles within the asphalt matrix, and DSR tests were conducted to evaluate the high-temperature rheological performance of CRMA before and after filtration. GRA was further employed to identify the dominant factors influencing the high-temperature rheological performance of CRMA. The main conclusions are summarized as follows:

(1) The filtration weighing results show that $R_{\mathrm{cr}}$ increased with the increasing CR particle sizes and contents, suggesting a stronger particle-related contribution to the high-temperature performance of CRMA.

(2) FM imaging confirmed a generally uniform CR particle dispersion within the asphalt matrix, and showed that after filtration, only limited residual CR particles remained. Finer CR improved dispersion and higher CR contents promoted particle contacts and local agglomeration.

(3) CR modification markedly increased G* over 58–76 °C, indicating enhanced high-temperature deformation resistance, and G* decreased as the temperature increased; at a given CR content, G* increased with coarser CR particles, and the filtration markedly decreased G*, confirming that the enhancement is primarily influenced by PE, with an additional contribution from IE. Moreover, increasing CR contents at a given particle size further strengthened G* with increased PE and IE.

(4) *δ* decreased as CR particle size increased, indicating an enhanced recoverable deformation capacity of CRMA at a high temperature; increasing the CR content further decreased *δ* and improved the high-temperature viscoelastic response. After filtration, *δ* increased markedly but remained lower than that of the base asphalt, indicating that the improvement in recoverable deformation capacity is dominated by PE, with IE providing additional gains, particularly at higher CR contents.

(5) $R_{\mathrm{cr}}$ correlated positively with G* and negatively with *δ*, and GRA indicated that CR content acts as the primary factor and CR particle size acts as the secondary factor for the high-temperature performance of CRMA.

## Figures and Tables

**Table 1:** Technical properties of DH70 base asphalt.

Item	Measured	Specification	Test Method
Penetration (25 °C)/0.1 mm	66.6	60~80	T0604 [33]
Penetration Index	−0.83	−1.5~+1.0	T0604 [33]
Softening Point/°C	49	≥46	T0606 [33]
Ductility (15 °C)/cm	>100	≥100	T0605 [33]

**Table 2:** Technical properties of CR.

Item	Measured	Specification	Test Method
Residue on Sieve/%	6.6	<10	JT/T 797 [34]
Relative Density	1.15	1.1~1.3	
Fiber Content/%	0.06	<1	
Metal Content/%	0.008	<0.05	
Carbon Black Content/%	30	≥28	GB/T 14837.1 [35]

## Data Availability

The original contributions presented in this study are included in the article. Further inquiries can be directed to the corresponding authors.
